# Distinctive Patterns of Evolution of the *δ-Globin* Gene (HBD) in Primates

**DOI:** 10.1371/journal.pone.0123365

**Published:** 2015-04-08

**Authors:** Ana Moleirinho, Alexandra M. Lopes, Susana Seixas, Ramiro Morales-Hojas, Maria J. Prata, António Amorim

**Affiliations:** 1 Instituto de Investigação e Inovação em Saúde, Universidade do Porto, Porto, Portugal; 2 IPATIMUP–Institute of Molecular Pathology and Immunology, University of Porto, Porto, Portugal; 3 Department of Biology, Faculty of Sciences, University of Porto, Porto, Portugal; 4 Genetics and Genomics Group, The Pirbright Institute, Compton Laboratory, Compton, Berkshire, United Kingdom; Universitat Pompeu Fabra, SPAIN

## Abstract

In most vertebrates, hemoglobin (Hb) is a heterotetramer composed of two dissimilar globin chains, which change during development according to the patterns of expression of α- and β-globin family members. In placental mammals, the β-globin cluster includes three early-expressed genes, ε(*HBE*)-γ(*HBG*)-ψβ(*HBBP1*), and the late expressed genes, δ (*HBD*) and β (*HBB*). While *HBB* encodes the major adult β-globin chain, *HBD* is weakly expressed or totally silent. Paradoxically, in human populations *HBD* shows high levels of conservation typical of genes under strong evolutionary constraints, possibly due to a regulatory role in the fetal-to-adult switch unique of Anthropoid primates. In this study, we have performed a comprehensive phylogenetic and comparative analysis of the two adult *β-like globin* genes in a set of diverse mammalian taxa, focusing on the evolution and functional divergence of *HBD* in primates. Our analysis revealed that anthropoids are an exception to a general pattern of concerted evolution in placental mammals, showing a high level of sequence conservation at *HBD*, less frequent and shorter gene conversion events. Moreover, this lineage is unique in the retention of a functional GATA-1 motif, known to be involved in the control of the developmental expression of the *β-like globin* genes. We further show that not only the mode but also the rate of evolution of the *δ-globin* gene in higher primates are strictly associated with the fetal/adult β-cluster developmental switch. To gain further insight into the possible functional constraints that have been shaping the evolutionary history of *HBD* in primates, we calculated dN/dS (ω) ratios under alternative models of gene evolution. Although our results indicate that *HBD* might have experienced different selective pressures throughout primate evolution, as shown by different ω values between apes and Old World Monkeys + New World Monkeys (0.06 versus 0.43, respectively), these estimates corroborated a constrained evolution for *HBD* in Anthropoid lineages, which is unlikely to be related to protein function. Collectively, these findings suggest that sequence change at the *δ-globin* gene has been under strong selective constraints over 65 Myr of primate evolution, likely due to a regulatory role in ontogenic switches of gene expression.

## Introduction

Hemoglobin (Hb), found in the circulating red blood cells of all vertebrates, is the major oxygen-transporting molecule, playing a key role in the cellular aerobic metabolism [27]. In mammals, Hb is a heterotetramer composed of two α-like and two β-like globin chains that are differentially expressed during development, such that functionally distinct Hb isoforms are synthesized in embryonic and adult erythroid cells [27–29].

These globin chains are encoded by members of the *α-* and *β-globin* gene families, which arose via tandem duplication of an ancestral, single-copy globin gene approximately 450–500 Mya, in the common ancestor of jawed vertebrates [14,23,25,35,78]. The two paralogous gene families exhibit a number of significant differences in gene content among jawed vertebrate taxa. These differences are especially pronounced in the case of the *β-globin* cluster, in which distinct repertoires of mammalian *β-like globin* genes originated by independent lineage-specific duplications followed by functional divergence [33–35,52–54,57,78,79]. In both monotremes and marsupials, the *β-globin gene* cluster contains a single pair of genes, the early expressed ε-globin and the late expressed *β-globin* [53]. In contrast, within the eutherian stem, further tandem duplications gave rise to a cluster of five *β-like globin* genes, containing early-expressed genes, located at the 5’ end of the cluster ε-(*HBE*)-γ(*HBG*)-ψβ(*HBBP1*), and late expressed genes, δ (*HBD*) and β (*HBB*), at the 3’ end, consistent with the orientation in contemporary species [26,31,53]. The fine tuning of the level and timing of expression of each of these genes relies on interactions with the locus control region (LCR), located from approximately 6 to 18 kb upstream of *HBE* [4,11,84].

Over the course of eutherian evolution the structure of the *β-globin gene* cluster has been dynamic and the late-expressed *HBD* and *HBB* paralogs have experienced different evolutionary fates. In the majority of mammals, the adult form of Hb (α_2_β_2_) contains similar β-chain subunits which are encoded by one or more copies of the *HBB* gene [52]. Contrastingly, the *δ-globin* gene, although present in almost all eutherian species examined to date, is frequently pseudogenized [19,24,26,30,54]. In a few species, a transcriptionally active but weakly expressed copy of the *δ-globin* gene was maintained, encoding the δ-globin chain of the minor fraction of the adult Hb (α_2_δ_2_), known as HbA_2,_ which is thus assumed to be physiologically irrelevant [45,46,76]. In fact, the δ-globin chain is absent in Old World Monkeys (OWM) [45,46] and ranges from 1% concentration in hominoids [10] to 40% in the galago [80], reaching 6% in New World Monkeys (NWM) [74] and 18% in tarsiers [40]. Surprisingly, in some eutherians *HBD* shows a level of sequence conservation typical of genes under strong evolutionary constraints [91]. In humans for example, *HBD* was found to have lower nucleotide diversity than *HBB*, suggesting that purifying selection has shaped the evolutionary history of *HBD* [49,87] through an unrecognized role not associated with oxygen transport [46,74,86].

The involvement of *HBD* in the fetal/adult Hb switch was proposed decades ago [5,55] and since then some studies have provided evidence supporting this hypothesis [6,49,67]. In fact, the fetal to adult Hb switch of anthropoid primates is unique. Furthermore, while both Anthropoids and Prosimians possess a γ-globin gene, its switch after birth only takes place in the major anthropoid branch, the catarrhines, occurring earlier in NWM, whereas in Prosimians it is only expressed at the embryonic stage [38]. Therefore, phylogenetic and comparative genomic analysis across placental mammals with distinct repertoires of β-like genes and corresponding expression programs should provide clues to the evolution and putative functional divergence of the *δ-globin* gene.

However, the evolutionary history of the eutherian *HBD* is quite complex due to unusually frequent sequence exchanges through extensive gene conversion and unequal recombination with its neighbor, *β-globin* [19,31,40,54,80], resulting in extensive sequence homogenization and hampering the assignment of orthologous relationships among *HBD* and *HBB* genes. Indeed, *HBD* was initially thought to be the result of a recent duplication in primate evolution, approximately 40 MY [18], but recently an older origin has been proposed [30,53]. Under such scenario of controversy, a revisit of the evolutionary history of the adult *δ-globin* gene can help elucidate the origin of this gene family, which in spite of many efforts is not yet fully understood [31,37,40,45,46,53,60,74,80].

Here, we perform a comprehensive phylogenetic and comparative analysis of the two adult *β-like globin* genes in a wide range of mammalian taxa, with a special focus on primates. Our results further document reticulation in the topology of the evolutionary history of *δ-globin* gene, demonstrating that it has behaved as an evolutionary palimpsest, with repeated and partially overlapping β / δ sequence transfers obscuring orthology. Additionally, we show that the *δ-globin* gene is highly conserved in Anthropoids, with a particularly strong signal of purifying selection in Great Apes. Sequence conservation at this locus is unlikely related to protein function and may reflect mutational constraints on regulatory regions involved in the fetal-to-adult developmental expression switch of the *β-globin* cluster.

## Materials and Methods

### DNA Sequence data and gene identification

To obtain DNA sequences spanning the entire *HBD* and *HBB* genes, we used Blat queries to interrogate the genome assemblies of several mammalian species available in the UCSC Genome Browser website (http://genome-euro.ucsc.edu/index.html). Whenever the *HBB* sequence was available, we used it to identify its paralogous sequences (the first hit); alternatively we used the human *HBB* and *HBD* sequences to identify their corresponding orthologs. Due to a history of concerted evolution, in some cases high sequence identity between *HBD* and *HBB* produced ambiguous results. In these cases the genome coordinates were used to distinguish between the two genes, since the order of the *β-like globin* genes has been maintained throughout mammalian evolution. Additional genomic data was obtained either from the High Throughput Genomic Sequences database (HTSG), Trace Archives or by direct sequencing to complete sequence gaps and to include further mammalian species. Detailed information on sampling is listed in S1 Table. Following all these steps of manual curation, our sample included 29 sequences from the three major subclasses of mammals: 1 Prototheria (*Ornithorhynchus anatinus*), 1 Methateria (*Monodelphis domestica*) and 27 Eutheria, including representatives of the following superordinal groups (1 Xenarthra, 7 Laurasiatheria and 19 Euarchontoglires). We also included one avian species (*Gallus gallus*) as outgroup.

### Evolutionary analysis

The reconstruction of the evolutionary history of the *HBD* and *HBB* genes was carried out across the mammalian phylogenetic tree with the chicken sequence as outgroup. The identified coding sequences were translated into the corresponding protein and aligned using the Expresso mode of T-Coffee in order to take into account any structural information of the protein available in the databases [2]. Each of the non-coding sequences, including the 5’ and 3’ untranslated regions (UTRs) plus 2 introns, were aligned separately as nucleotide sequences using the accurate mode of T-Coffee [51]. Phylogenetic reconstruction was performed using Maximum Likelihood (ML) and Bayesian Inference (BI) as the optimality criteria. Trees were estimated using the complete gene sequence and including only the coding sequence (CDS). The partition strategies and models of evolution implemented in these analyses were identified using PartitionFinder v 1.1.1 [41]. PartitionFinder was run specifying the raxml and mrbayes models of evolution for computational capacity reasons.

ML analyses were performed with RAxML [75] run in the CIPRES Science Gateway [48]. Phylogenetic analysis of the complete gene was run with the following partitions: 1) 5’ UTR + Exon 1, 2 and 3 + Intron 1; and 2) Intron 2 + 3’ UTR. The GTR+G+I model was implemented for each of these. ML analysis of the CDS was performed with three partitions corresponding to the three codon positions. The GTR+G model was implemented for each partition. The resulting trees were evaluated with 1000 bootstrap replicates.

BI analyses were run in MrBayes 3.2 [64] in the CIPRES Science Gateway. The complete gene analysis was performed with three partitions: 1) 5’ UTR + Exon 1, 2 and 3 + Intron 1; 2) Intron 2; and 3) 3’ UTR. The implemented models of evolution were the K80+G, GTR+G+I and HKY+G+I for the first, second and third partitions, respectively. BI with the CDS sequence was performed with the data matrix partitioned according to codon position; the models of evolution implemented were the K80+G for the first codon position and the GTR+G for the second and third positions. Two independent runs of 10 million (complete gene analysis) and 5 million (CDS analysis) generations with 8 chains each (7 heated and one cold) were set up. Trees were sampled every 200 (complete gene) or 100 (CDS) generation and the first 12500 trees (25% of the sample) were discarded as burn-in. Convergence and burn-in were assessed using Tracer 1.6 [61], MCMC Trace Analysis Package http://tree.bio.ed.ac.uk/software/tracer/).

Additionally, a third phylogenetic analysis was performed using models of codon substitution with the coding sequence only as input. This method allows us to take into consideration any potential divergence in codon usage across the mammalian lineage and differences in selective pressure among gene copies, which included pseudogenised copies. Analyses were run using CodonPhyML [21] with the GY CF3x4 [22] model of codon substitution and specifying the M3 model of selective pressure [90]. An initial tree was obtained using BioNJ + GYECMK07 and the topology was searched using the subtree pruning and regrafting (SPR) heuristic search. Branch support was obtained using the approximate Likelihood Ration Test (aLRT) as implemented in the software. To identify potential recombination events in our primate data set we used the recombination detection package (RDP3) [43], applying a set of seven statistical methods, which includes RDP [42], GENECONV [56], Bootscan [44], Maxchi [72], Chimaera [59], SiSscan [20] and 3Seq [8]. Briefly, two of these are phylogenetic methods, which infer recombination when different parts of the genome result in discordant topologies (RDP and Bootscan), while the other five are nucleotide substitution methods, which examine the sequences either for a significant clustering of substitutions or for a fit to an expected statistical distribution: MaxChi, Chimaera, Geneconv, 3Seq and SiScan. The latter primarily uses genetic similarity estimates but also takes some phylogenetic information into account. To uncover both species specific and ancient events of gene conversion, we conducted the analysis with the two paralogous genes for all species simultaneously. Settings for all methods executed in RDP3 were as follows: Sequences were considered to be linear, the p-value cutoff was set to 0.05, and the standard Bonferroni correction was used. In addition, phylogenetic relationships were recovered for each fragment showing signs of gene conversion, in Anthropoids and Catarrhines, following the same procedure described above. ML analyses were performed using the GTR+G model without sequence partitioning. The resulting trees were evaluated with 1000 bootstrap replicates.

### Search for open reading frames and promoter analysis

The genomic sequence of the *HBB* and *HBD* genes was used to predict the locations and exon-intron structures using the program Genscan [12], followed by sequence alignment of known exon sequences and manual inspection of homology. Protein sequence was obtained using the translation tool implemented in the ExPASy web server (http://www.expasy.org) [3]. Sequence alignment for eutherian *HBB* proteins and *HBD* open reading frame were performed using ClustalW [83] implemented in Geneious version 5.5 created by Biomatters (available from http://www.geneious.com/). To identify conserved motifs previously shown to be essential for *HBB* and *HBD* expression, promoter sequences located 5’ to the two *β-like globin* genes (~200bp) were aligned using the same approach as above. To confirm transcription factor binding site matches identified within the alignments, we used MatInspector implemented in the Genomatix Software Suite (http://www.genomatix.de/index.html).

### Evolutionary rate estimates and selection tests

Pairwise sequence divergence was deduced from Jukes and Cantor distance calculated with DnaSP v.5.10 [65]. In our estimates we scored each insertion or deletion, regardless of length, as one difference as in [13]. Divergence times between species were obtained with TimeTree [32]. Maximum-likelihood estimates of dN/dS (ω; dS—synonymous substitution rate and dN—non-synonymous substitution rate) were carried out using the codeml program from the software package Phylogenetic Analysis by Maximum Likelihood—PAML version 4.8 [89]. To investigate the selective pressures that have shaped the evolution of *HBB* and *HBD* genes we first calculated dN/dS ratios (M0 model) for each gene separately in the entire mammalian phylogeny. Next, to test the hypothesis of variable selective pressures among *HBD* in primates, we performed nested branch models using either the one-ratio model calculated for the whole anthropoid phylogeny, the two-ratio estimated for Great Apes and other primates and three-ratio inferred for Great Apes, OWM and NWM [7,88]. Although ω values below 1 (ω < 1) are generally considered as an evidence of purifying selection, to reject the hypothesis of neutral evolution all models were compared with a null model where ω was fixed to 1 (ω = 1). The significance was obtained with likelihood ratio tests (LRT) which were calculated as twice the variation of the likelihoods (-2Δ*l*) with a χ^2^ distribution. For the calculation of *HBD* ω values, pseudogene sequences were only included after the removal of positions affected by premature stop codons and frameshift mutations. In the specific case of lemur species, the *HBD* sequences were excluded from the analysis given their hybrid *ψβ/δ* nature [37].

## Results

### Evolutionary history of the two adult β-like genes in mammals

**Fig 1:**
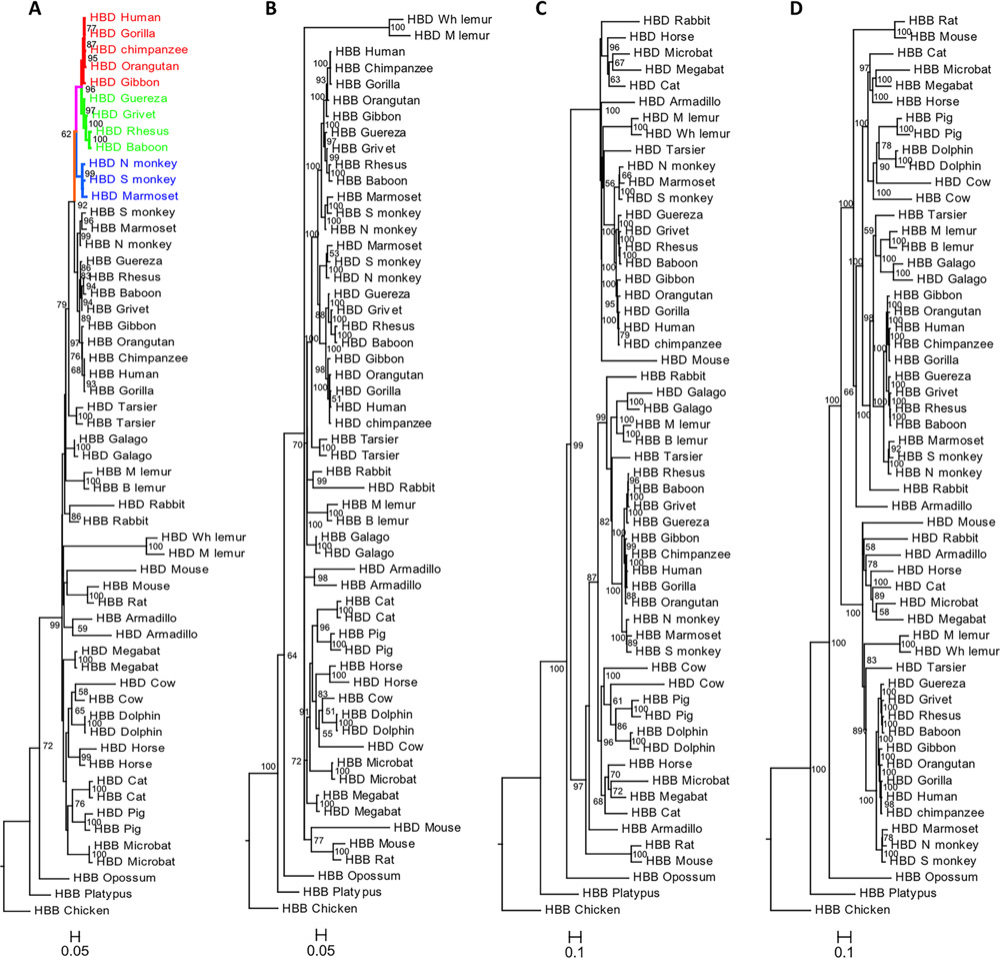
Phylograms depicting relationships among adult *β-like* genes in mammals. The phylogeny reconstructions were performed using two methods: A), C) Maximum Likelihood and B), D) Bayesian Inference; trees were estimated using the A), B) coding sequence and C), D) complete gene sequence. Branch support values are given on the internodes. Red branches represent the Great Apes, green the OWM and blue the NWM; the pink and orange branches represent the common branch of Catarrhines and Anthropoids, respectively.

We conducted a phylogenetic analysis of the adult *β-like globin* genes, *HBD* and *HBB*, in a diverse dataset, including monotremes, marsupials and placental mammals, and one avian species, which was used as outgroup. The phylogenies obtained with ML, BI and the codon model approach were similar when either the complete gene sequence or the CDS were used (Fig 1A–1D and S4 Fig).

However, trees obtained using the complete gene were different from those estimated with the CDS only (Fig 1 and S4 Fig). In the phylogenies based on the CDS the two *β-like* paralogous genes from the same species cluster together, consistent with a process of interparalogue gene conversion, referred to as “concerted evolution” and previously described in several taxa [19,31,40,52,80]. The exceptions to this general pattern occur in Anthropoid primates (monkeys and apes) where *HBD* and *HBB* are grouped into two reciprocally monophyletic groups, and in the lemur species. This could lead to the erroneous interpretation that *HBD* arose recently through a duplication in primate evolution, as initially thought [18], but more likely reflects a gene conversion event in the common ancestor of those primate lineages. In the lemur species, the exception is easily explained by the fact that in the ancestry of lemurs a hybrid ψβ/δ pseudogene was created by unequal crossing-over between misaligned *HBD* and *HBBP1* sequences [37]. Finally, an *HBD* ortholog is absent from rat, and in mouse we found no evidence for interparalog gene conversion between *HBD* and *HBB* orthologs (*HBD-T1* and *HBB-T1*), in agreement with previous results [33]. Phylogenetic analyses based on the entire gene sequence lead to better-supported trees, which more reliably replicate the species tree, recovering the true evolutionary history of gene duplication. This phylogeny is consistent with previous results supporting a duplication event of *HBB* and *HBD* genes after the marsupial/eutherian split [53]. Both ML and BI trees contain two major sister clades, one comprising the *HBD* gene copies of most species and a second clade mainly with the *HBB* genes. Within the *HBB* clade, the *HBD* genes from galago, cow, pig and dolphins are clustered with each of their *HBB* paralogs. In galago, it is well documented that the ancestral *HBD* gene was almost completely converted by the *HBB* gene, explaining the monophyletic pattern observed [80]. According to a recent analysis the phylogenetic incongruence seen in the latter three species is likely due to independent gene conversion events that followed an extensive unequal cross-over spanning *HBD* intron 2 in the stem lineage of cetartiodactyls [19]. Our results also confirm that the interparalog conversion is largely restricted to the coding regions of globin genes [19,26,30,31,40,45,53], because the incorporation of the phylogenetic information of non-coding sequence, including intronic and 5’ and 3’ flanking sequence, proved to be especially useful in assigning orthologous relationships.

### Analysis of gene structure

In all species examined the *HBB* gene retains an intact ORF with conserved donor/acceptor splice sites encoding a polypeptide of 144–146 amino acids (S1 Fig). By contrast, the *HBD* gene has accumulated several inactivating mutations throughout the mammalian phylogeny. These include the introduction of premature stop codons, small insertions and deletions and mutations in the consensus donor (GT) or acceptor (AG) splice sites (S2 Fig). Moreover, *HBB* and *HBD* also display different across species conservation patterns (S3 Fig). The *HBB* promoter is highly conserved and contains conserved consensus TATA, CAAT and EKLF (Erythroid Krüppel-like factor) binding motifs in most species examined. Only cat and cow show substitutions in the EKLF consensus sequence (S3A Fig) but present an upstream EKLF binding element, identified by MatInspector, which is likely to replace the possible disrupted EKLF motif. A lower conservation in *HBD* promoter region is readily apparent from the difficulty in obtaining a good multiple alignment for all species. We detected three species, galago, cat and microbat, in which *HBD* has a β-like promoter, acquired through independent gene conversion events (S3A Fig), as previously shown [19,80]. In anthropoids, high sequence homology was observed at the *HBD* promoter region (S3B Fig), with a conserved consensus TATA binding motif, a functional GATA-1 motif [47] close to the mutated CAAC box, and lack of the EKLF binding element in all these species. These features of the *HBD* promoter region have been shown to be responsible for the low expression of the adult *δ-globin* gene [63,81,82]. The remaining species (S3C Fig) all share the major defect in the proximal δ-promoter, the absence of a consensus EKLF-binding motif, but do not have the GATA-1 motif common to all other δ-like promoters. The lack of various conserved motifs in the *HBD* promoters that are crucial for β-like globin expression suggests that they are transcriptionally inefficient in tarsier, mouse, rabbit, dolphin, cow, pig, horse, megabat and armadillo.

### Recombination events in primates

In the phylogenetic analyses we did not detect evidence of recombination events between *HBD* and *HBB* in anthropoid primates. This result is in agreement with other studies that proposed a δ-β gene conversion in the anthropoid stem [40]. However, it has been suggested that further gene conversions occurred independently in catarrhine and platyrrhine lineages [37,45,60]. Although we did not find phylogenetic evidence for gene conversions within these primate lineages, we cannot exclude the possibility of short-tract gene conversion events not detectable by phylogenetic analysis. Therefore, we sought to re-examine the possibility of lineage-specific gene conversion taking advantage of a more comprehensive sample of primate species and the use of multiple methods implemented in the software package RDP3 [43] for detecting recombination signals and putative recombinant sequences. We found robust signals for four independent interparalog gene conversion events, in which portions of *HBB* were copied onto *HBD* (summarized in Table 1).

**Table 1:** Summary of gene conversion analysis for primate *HBD* and *HBB* paralogues.

Gene conversion event ID				Breakpoint ^a^ Positions		Conversion Tract Length	Detection Methods ^a^	P-value ^a^
	Recombinant sequences	Major Parental Sequence ^b^	Minor Parental Sequence ^b^	Begin	End			
**1**	HBD_Tarsier	unknown	HBB_Tarsier	96	574	72 bp 5’ flanking -183 bp into exon 2	RDP, **GENECONV**, BootScan, MaxChi, Chimaera, SiScan, 3Seq	3,475 x 10^-25^
**2**	HBD_Galago	unknown	HBB_Galago	12	2036	149 bp 5’ flanking—55 bp into exon 3	RDP, GENECONV, BootScan, MaxChi, Chimaera, SiScan, **3Seq**	3,665 x 10^-18^
**3**	HBD_Human HBD_Chimpanzee HBD_Gorilla HBD_Gibbon HBD_Guereza HBD_Grivet HBD_Rhesus HBD_Babbon	unknown	HBB_Babbon	97	385	71 bp 5’ flanking -123 into intron 1	RDP, **MaxChi**, Chimaera	3,198 x 10^-4^
**4**	HBD_Human HBD_chimpanzee HBD_Gorilla HBD_Gibbon HBD_Guereza HBD_Grivet HBD_Rhesus HBD_Babbon HBD_N.monkey HBD_Marmoset HBD_S.monkey	unknown	HBB_N.monkey	197/367 ^c^	632	106 bp 5’ flanking -215 bp into exon 2	RDP	1,115 x 10^-5^

^a^ In cases where multiple methods detected the same or a similar conversion event, we reported the breakpoint positions and the method yielding the lowest average Bonferroni corrected p-value, which is shown in bold; breakpoint positions refer to the nucleotide positions in the full alignment of the Primate *HBB* and *HBD* sequences.

^b^ The major parental and minor parental sequences correspond to the parent contributing to the larger fraction and to the minor fraction of the recombinant sequence, respectively. In all 4 events the major parental sequence is unknown given that the presence of a parent and a recombinant in the alignment is sufficient for a recombination event to be detected by these methods.

^c^ The breakpoint for the recombination event number 4 varies depending on the species in which it was detected: 197 in Platyrrhines (N.monkey, Marmoset and S.monkey) and 367 in Catarrhines (Human, Chimpanzee, Gorilla, Gibbon, Guereza, Grivet, Rhesus, Babbon), leading to different gene conversion tract length predictions for these groups.

Two of these gene conversion events, 1 and 2, were detected by all seven methods, and corroborate independent gene conversions previously identified in tarsier and galago [40,80]. A third event was detected, corresponding to the conversion of the first *HBD* exon and intron by *HBB* sequences, in catarrhines (OWM and Great Apes). Since this event was detected in orthologous *HBD* copies of all catarrhines represented, it most likely took place before the divergence of OWM and Great Apes, along the pink branch in Fig 1A. The event number 4 corresponds to a more extensive conversion tract present in all anthropoids (platyrrhines and catarrhines), suggesting that those sequences have all descended from an anthropoid ancestor sequence in which the recombination event occurred (orange branch in Fig 1A). This δ-β conversion appears to extend from the 5’ promoter region till the end of the second exon, however in catarrhines the signal for this older gene conversion is restricted to the second exon, because a subsequent event (identified as number 3) overprinted part of the older one.

In order to assess whether the conversion events 3 and 4 have been correctly identified, we constructed and compared two phylogenetic trees, one with the region containing evidence for an older gene conversion in both platyrrhines and catarrhines (nucleotide 367–632), and a second one with the portion of the alignment between the inferred breakpoints in event 3 (nucleotide 97–385) where a second, more recent conversion event occurred in the catarrhine stem (S5 Fig). The topology of the first tree (S5A Fig) is consistent with a gene conversion in the common ancestor of Anthropoids, in agreement with results obtained with other methods [31,40,45,73]. In contrast, in the second tree (S5B Fig) the *HBD* and *HBB* genes group together within the catarrhine and platyrrhine lineages, as expected under the hypothesis of a conversion event restricted to catarrhines. This tree topology could also indicate that parallel gene conversion events have occurred in the stem of catarrhines and platyrrhines; however, no gene conversion event in platyrrhines has been detected with any of the methods used in our analysis. Nevertheless, this alternative hypothesis remains disputable given that an older conversion event occurring between still very closely related sequences would be very difficult to detect. Therefore, although our results do not fully support previous evidence that *HBD* has been involved in a conversion in platyrrhines [60], it cannot be ruled out. It is noteworthy that albeit two independent gene conversion events occurred in different Anthropoid lineages, the GATA-1 motif in the *HBD* promoter region remained intact.

### Evolutionary rates and functional constraints in primates

**Fig 2:**
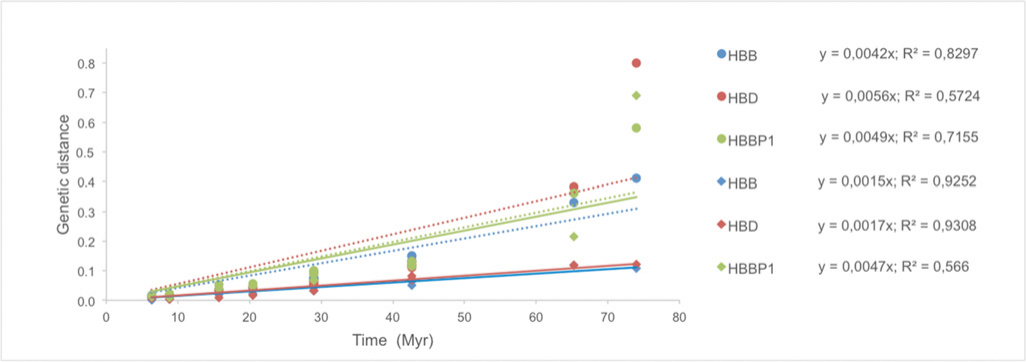
Genetic distance vs divergence times between human and different primate species (Anthropoids and Prosimians) for *β-like* genes. Circles and dotted lines correspond to introns while diamonds and solid lines correspond to exons. Divergence times between humans and other species were obtained with TimeTree [32] and are as follows: Ptr: 6,3 Myr; Ggo 8,8 Myr; Ppy: 15.7 Myr; Nle 20,4 Myr; OWM (Mcc, Panu, Cgue and Caa): 29 Myr; NWM (Sbol, Cjac and Anan): 42,6 Myr; Tsyr: 65,5 Myr; Ogar: 74 Myr. Linear regression trend lines were set to intercept the origin.

From our previous analysis, it is apparent that while *HBD* has diverged at markedly different rates in different primate lineages, as shown by the variable branch lengths in the phylogenetic trees (Fig 1), anthropoid *HBD*s share a high sequence identity not only in their coding region but also at the promoter. As a first measure of the rate of *HBD* evolution, we compared the genetic distance between humans and 13 other primate species, for both adult *β-like globin* genes. *HBBP1* was also included in the analysis due to the unusually slow substitution rates previously reported for this pseudogene estimated by comparison of human, gorilla and chimpanzee sequences [13]. Genetic distances were then plotted against the corresponding divergence times for each pairwise comparison, and the linear regression trend line was estimated for each group, as shown in Fig 2.

From the slope of the trend lines and the r^2^ values we are able to compare the rate of intron and exon evolution and its constancy over time, respectively. The results presented in Fig 2 and S2 Table show that, overall, exons evolved at a lower rate than introns, except for *HBBP1*, in which nucleotide differences are more homogeneously distributed between exons and introns, as expected for a pseudogene. In Prosimians, there is a trend towards increasing evolutionary rates, more pronounced in non-coding regions (introns and *HBPP1* exons). Remarkably, the rate of evolution of *HBD* exons has remained relatively constant across primate evolution (r^2^ = 0.95) and is comparable to that of *HBB* exons, even though in higher primates *HBD* is either silent or contributes to only a very small fraction of adult Hb. To gain further insight into the possible functional constraints that have shaped the evolutionary history of *HBD* in primates, we calculated dN/dS (ω) ratios under alternative models of gene evolution (table 2).

**Table 2:** Parameter Estimates and Likelihood Scores under Different Branch Models.

Model	Parameters for branches	Likelihood (*l*)
One ratio	ω_*HBB*_ = 0.26**	-4397.16
	ω_*HBD*_ = 0.40**	-3509.38
	ω_*HBD*_Anthropoids_ = 0.31**	-1019.71
Two ratio	ω_*HBD*_Apes_ = 0.06**	-1015.62
	ω_*HBD*_OWM+NWM_ = 0.43*	
Three ratio	ω_*HBD*_Apes_ = 0.06**	-1015.45
	ω_*HBD*_OWM_ = 0.53	
	ω_*HBD*_NWM_ = 0.38*	
**Models Compared**	**-2Δ*l***	
One vs. Two ratios	8.18 (df = 1) *	
Two vs. Three ratios	0.34 (df = 1)	

NOTE— ω_*HBB*_ and ω_*HBD*_, ω for all *HBB* and *HBD* lineages, respectively; ω_*HBD*_Anthropoids_, ω for all Anthropoid *HBD* lineages; ω_*HBD*_Apes_, ω for Great ape *HBD* lineages; ω_*HBD*_OWM+NWM_, ω for all OWM and NWM *HBD* lineages; ω_*HBD*_OWM_ and ω_*HBD*_NWM_, ω for OWM and NWM *HBD* lineages, respectively; df—degrees of freedom.

*Significant P < 0.01

**Significant P < 0.001

First, we estimated single ω values for the entire mammalian phylogeny (M0 model), for either *HBB* or *HBD* genes. The observed ω values were significantly lower than 1 (ω_*HBB*_ = 0.26 and ω_*HBD*_ = 0.40) pointing to an overall conservation of *HBB* and *HBD*. The ω obtained for *HBB* is the expected for a functional gene and is in agreement with previous estimates [1]. In the case of *HBD* orthologs, the detected signal of purifying selection may be an outcome of gene conversion by the *HBB* gene in multiple lineages, mostly in the coding region. As illegitimate recombination between *HBD* and *HBB* has been noticeably reduced since the last common ancestor of Anthropoids 65.5 Mya, we examined the extent of the selective pressures exerted in this specific clade. Overall, the significance of the low value obtained under the one-ratio model (ω_*HBD*_Anthropoids_ = 0.31) rejects the hypothesis of a neutral evolution of *HBD* in anthropoids. Then, to examine whether *HBD* has been subject to variable selective constraints among different anthropoid clades (Fig 1A), we applied the two-ratio model separating the phylogenetic group of Great Apes from all remaining primates (OWM and NWM). In addition, we also applied the three-ratio model, in which ω was allowed to vary across Great Apes, OWM and NWM clades. The comparison of the one-ratio and the two-ratio models showed that the two-ratio presents an improved fit to anthropoid *HBD* evolution and that it did not differ significantly from the three-ratio model. Nevertheless, in both two-ratio and three-ratio models the Great Apes clade showed an extremely low ω value (ω_*HBD*_Apes_ = 0.06), while the OWM+NWM branches present a higher ω (ω_*HBD*_OWM+NWM_ = 0.43), but still suggesting a constrained evolution. Although our results indicate that *HBD* might have experienced different selective pressures throughout primate evolution, these estimates corroborated a high conservation of *HBD* in Anthropoid lineages that is unlikely related to protein function, since in most primate species this gene is either weakly expressed or not transcribed at all.

## Discussion

In humans and in chimpanzees, unusually high levels of *HBD* sequence conservation, when compared to functional paralogs, have been described [49]. Such pattern of conservation has been difficult to reconcile with the negligible expression of HbA_2_. Moreover, the evolutionary history of *HBD* is complex and orthologous relationships among *HBD* and its paralog gene (*HBB*) have been obscured by a history of recurrent gene conversion and unequal crossing overs, throughout eutherian evolution [19,52–54]. Here we gained insight into the evolutionary history of *HBD* and its likely regulatory role in the fetal-to-adult switch unique of Anthropoids, by performing a comprehensive phylogenetic and comparative analysis of the two adult *β-like globin* genes in a wide range of mammalian taxa. The results from our phylogenetic reconstruction are in agreement with previous findings which demonstrated that *HBD* duplication occurred before the radiation of Eutheria [53]. The obtained tree topology is also consistent with a history of concerted evolution between *HBD* and *HBB* that has created chimeric β/δ fusion genes in multiple, independent lineages [19,31,40,54,80]. However, our results show that primates represent an exception to this common trend, given that phylogenetic relationships are maintained throughout this lineage, suggesting that illegitimate recombination between *HBD* and *HBB* has been noticeably reduced since the last common ancestor of Anthropoids 65,5 Mya. Indeed, the recombination analyses here presented demonstrate that even though a certain level of gene conversion has occurred in some Anthropoid lineages, these events have taken place in the stem of the major branches (platyrrhines and catarrhines), as previously inferred from smaller datasets [31,40,45,60]. Moreover, gene conversion in Anthropoids was restricted to shorter regions (exon and intron 1) than those most frequently identified in other species (exons 1, 2, and 3 and intron 1) [19,30,31,40,80]. The distinct phylogenetic patterns between Anthropoid primates and nearly all other species, obtained when using the coding sequence, reflect the relative extent of these events. Although most mammals retain both adult *β-like* gene copies, only the *HBB* gene is functional and essential. *HBD* has apparently become dispensable and in several lineages has pseudogenized. In fact, we confirmed that *HBD* inactivation occurred in a wide range of eutherian species by the accumulation of loss-of-function mutations that disrupted either the ORF or the promoter region. It is noteworthy that when considering extant mammalian species it is only possible to establish bona-fide homology relationships between *HBD* orthologs among Anthropoid primates. Moreover, evolutionary tests suggest that *HBD* is evolving under selective constraints across different Anthropoid species, as hypothesized several decades ago [13,24,37,74]. Such high level of conservation is at odds with the variable expressivity of the δ-globin chain, which is absent in OWM [45,46] and ranges from 1% concentration in hominoids [10] to 6% in NWM [74]. Selective constraints on protein evolution do not seem a plausible explanation for the signal of purifying selection detected, since to date, *HBA*_*2*_ has no recognized physiological function [62,76,77].

Interestingly, a role for *HBD* and *HBBP1* in the regulatory mechanisms coordinating the fetal-to adult switch has been proposed in early independent studies [5,13,24,26,55]. Taking into account that the mechanism of Hb switch is common to all simian primates [38], we might expect to find similar patterns of conservation and diversity in ortholog *HBD* sequences for a 65.5 Myr time frame. Accordingly, the patterns of conservation we have now uncovered perfectly overlap with *HBG* duplication and the acquisition of a fetally expressed hemoglobin in anthropoid primates. Noteworthy, we detected in all anthropoid primates a conserved functional GATA-1 motif in the promoter of *HBD*, which has remained intact despite recurrent gene conversion events overlapping the promoter region among these lineages. Considering that *HBD* has very low expression levels in anthropoids, the conservation of a functional GATA-1 binding motif suggests other functional constraints rather than positive regulation of *δ-globin* gene expression. Indeed Gaudry, et al. [19] demonstrated that only late expressed *β-like globin* genes retaining an *HBB*-like promoter are efficiently transcribed. Developmental regulation of gene expression at the *β-globin* cluster involves the formation of chromatin loops mediated by several transcription factors and cofactors [66,68]. It has been shown that GATA-1, along with other cofactors, is required for efficient long-range chromatin interactions between LCR and *β-like globin* genes, namely at the time of γ- to β- globin switch [9,39,85]. Importantly, is has also been demonstrated that the *HBD* upstream region harbors a binding site for BCL11A, which is a biochemically validated and fundamental switching factor necessary for fetal hemoglobin silencing [67,69]. Remarkably, strong interactions between the LCR and the region encompassing both *HBD* and *HBBP1* were uncovered by chromosome conformation (3C and 5C) analyses at the *β-globin* locus [6,17,70]. Collectively, these findings suggest that *HBD* and *HBBP1* might be involved in chromatin looping in the human-globin cluster, a crucial mechanism for temporal coordination of gene expression [16,36]. Interestingly, the observed differences in the rate of evolution between the branches leading to Great Apes and the common branch of OWM and NWM suggest different selective pressures, which may reflect alternative mechanisms of controlling expression in the *β-globin* cluster among Anthropoids. Selective constraints on the protein function cannot be completely ruled out, although evidence of functional relevance of HbA_2_ is lacking. HbA_2_ has features that are nearly identical to those of HbA [15] but, even though in the absence of β-chain production in β-thalassemia major it becomes the predominant oxygen carrier, it never effectively replaces HbA function. Concordantly, in humans mutations in *HBD* are *per se* clinically silent [71,76]. The evolutionary history of *HBD* in mammals has been shaped by concerted evolution, however our results show that in the extant species of Anthropoids gene conversion events have not been frequent, and when they do occur the exchanged sequence tract is short. The low frequency of gene conversion as well as the conservation of a motif involved in chromatin remodeling could be an outcome of stronger selective constraints acting on *HBD* in anthropoids, as previously reported for other functionally important regions [50,58,92]. These results are also consistent with previous evidence of purifying selection reducing *HBD* genetic diversity in human populations and in chimpanzees [49]. We have now characterized the evolution of *HBD* in mammals and found that the unusual high levels of conservation in this genomic regions are shared across several primate species. In the light of recent advances in the understanding of the *β-globin* cluster regulation, we propose that the similar evolutionary trajectory of *HBD* in Anthropoids is due to functional constraints related with the intricate process of chromatin and protein interactions coordinating the developmental expression of β*-like globin* genes.

## Supporting Information

S1 Fig Sequence alignment for eutherian HBB proteins. (PDF)

S2 Fig Sequence alignment of eutherian *HBD* open reading frame. Blue and purple filled boxes mark the exons and donnor/acceptor splice sites, respectively. Dots represent nucleotide identities to the human sequence that was set as reference. Coloured nucleotides indicate changes to the human sequence and aminoacid alterations are marked by filled coloured boxes. The lemur species were excluded from the analysis given that their hybrid ψβ/δ pseudogene [37] generates multiple misalignments.
(PDF)

S3 Fig Sequence alignment of eutherian *HBB* and *HBD* promoters. A) *HBB* and *HBB*-like *HBD* promoters; B) Anthropoid *HBD* promoters and C) *HBD* promoters lacking the TF binding motifs which are conserved in *HBB*-like and *HBD*-like promoters. Conserved binding motifs are indicated in grey boxes. Again, the lemur species were excluded from the analysis.
(PDF)

S4 Fig Phylograms depicting relationships among adult *β-like* genes in mammals. The phylogenic tree, based on the coding sequence, was constructed using the Goldman–Yang codon model. Branch support values, obtained using the approximate Likelihood Ration Test (aLRT), are given on the internodes.
(PDF)

S5 Fig Maximum Likelihood phylograms depicting relationships among *β-like* genes of Anthropoids. The phylogeny reconstructions were based on A) the portion of the alignment that contain evidence of the anthropoid gene conversion (nucleotide 367–632) and B) the portion of the alignment between the inferred breakpoint in event 3 (nucleotide 97–385). Bootstrap branch support (1000 replicates) are given on the internodes.
(PDF)

S1 Table Listing of *HBB* and *HBD* sequences used for phylogeny reconstructions. (PDF)

S2 Table Evolutionary rates based on Jukes Cantor distance. (PDF)
