# Supplementary material for: Distinctive Patterns of Evolution of the δ-Globin Gene (HBD) in Primates
Source: PLoS One. 2015 Apr 8;10(4):e0123365. doi: 10.1371/journal.pone.0123365 (PMC4390247; doi:10.1371/journal.pone.0123365)
Supplement: S1 Fig — (PDF) [file pone.0123365.s001.pdf]

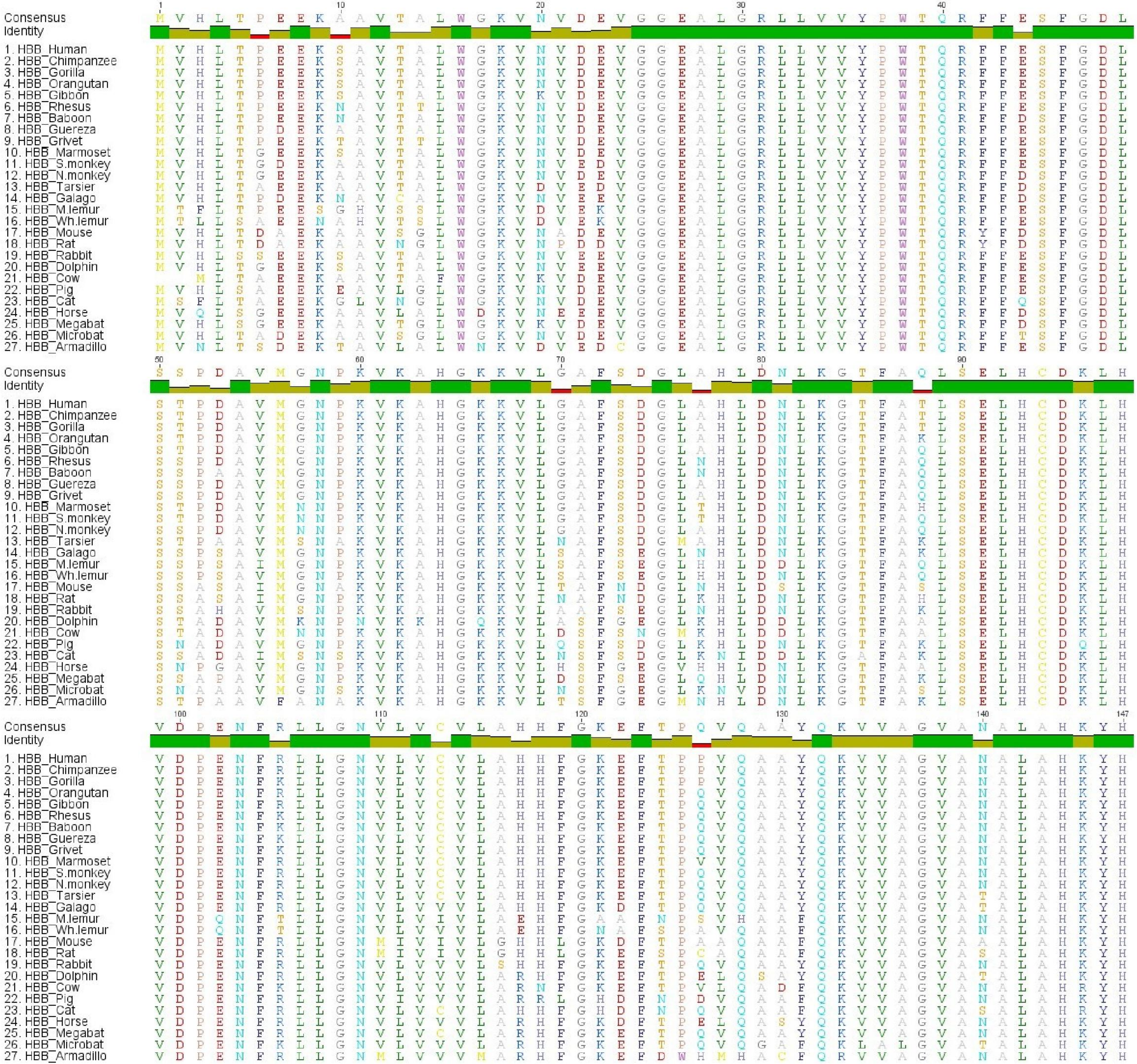

1 10 20 30 40
Consensus M V H L T P E E K A A V T A L W G K V N V D E V G G E A L G R L L V V Y P W T Q R F F E S F G D L
Identity
50 60 70 80 90
Consensus S S P D A V M G N P K V K A H G K K V L G A F S D G L A H L D N L K G T F A Q L S E L H C D K L H
Identity
100 110 120 130 140 147
Consensus V D P E N F R L L G N V L V C V L A H H F G K E F T P Q V Q A A Y Q K V V A G V A N A L A H K Y H
Identity
1. HBB_Human
2. HBB_Chimpanzee
3. HBB_Gorilla
4. HBB_Orangutan
5. HBB_Gibbon
6. HBB_Rhesus
7. HBB_Baboon
8. HBB_Guereza
9. HBB_Grivet
10. HBB_Marmoset
11. HBB_S.monkey
12. HBB_N.monkey
13. HBB_Tarsier
14. HBB_Galago
15. HBB_M.lemur
16. HBB_Wh.lemur
17. HBB_Mouse
18. HBB_Rat
19. HBB_Rabbit
20. HBB_Dolphin
21. HBB_Cow
22. HBB_Pig
23. HBB_Cat
24. HBB_Horse
25. HBB_Megabat
26. HBB_Microbat
27. HBB_Armadillo
