# Supplementary figures and images for: Distinctive Patterns of Evolution of the δ-Globin Gene (HBD) in Primates
Source: PLoS One. 2015 Apr 8;10(4):e0123365. doi: 10.1371/journal.pone.0123365 (PMC4390247; doi:10.1371/journal.pone.0123365)

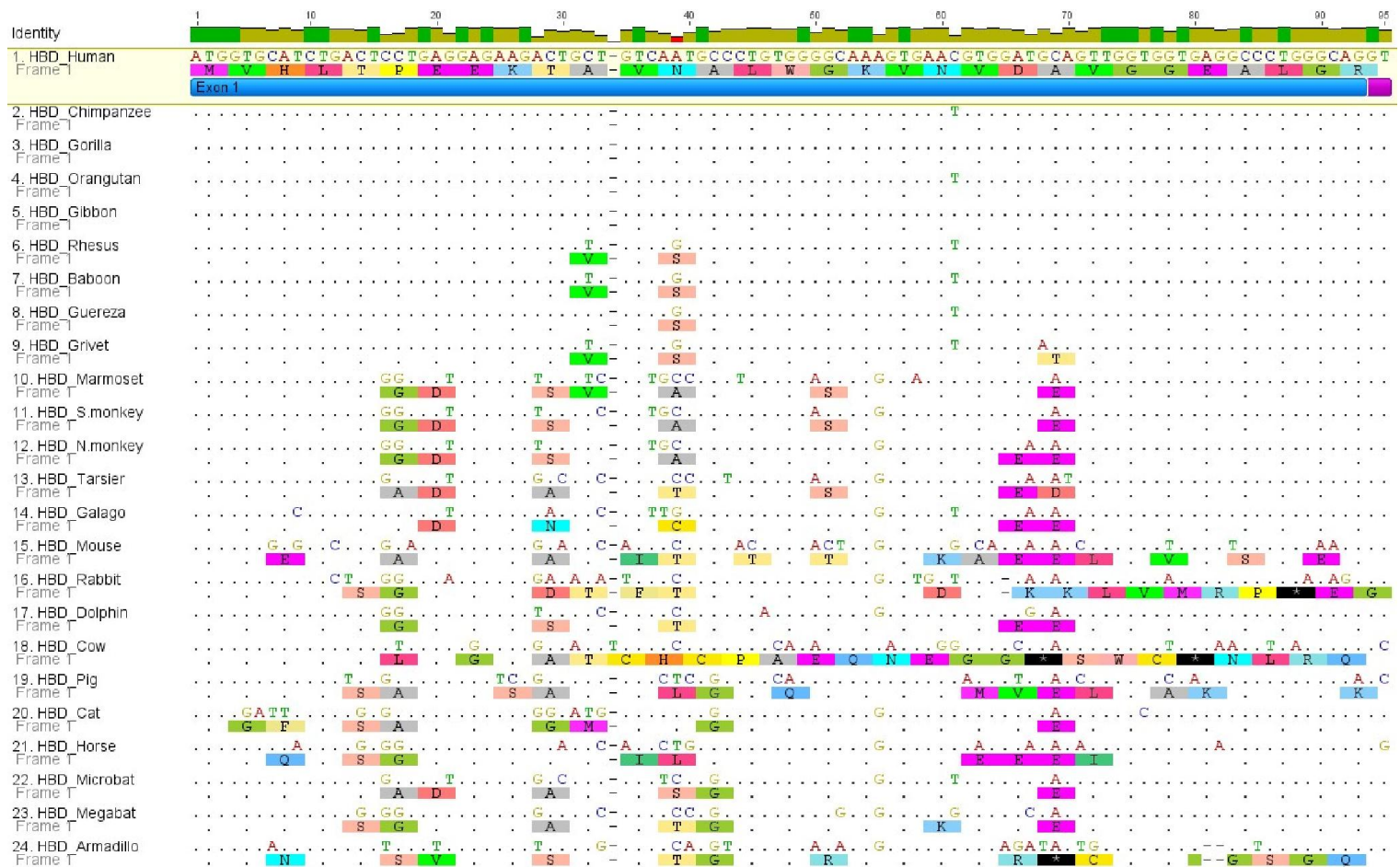

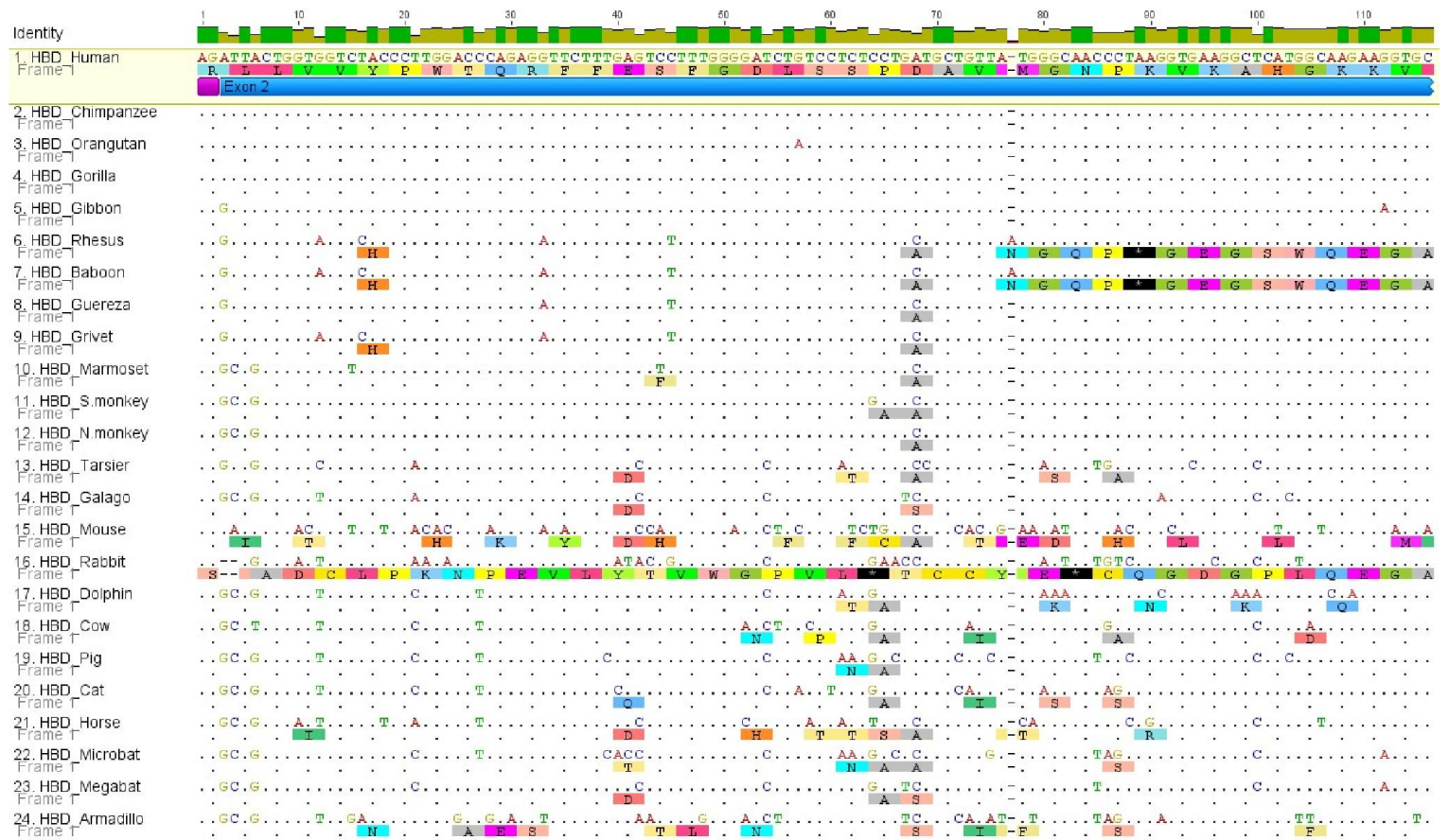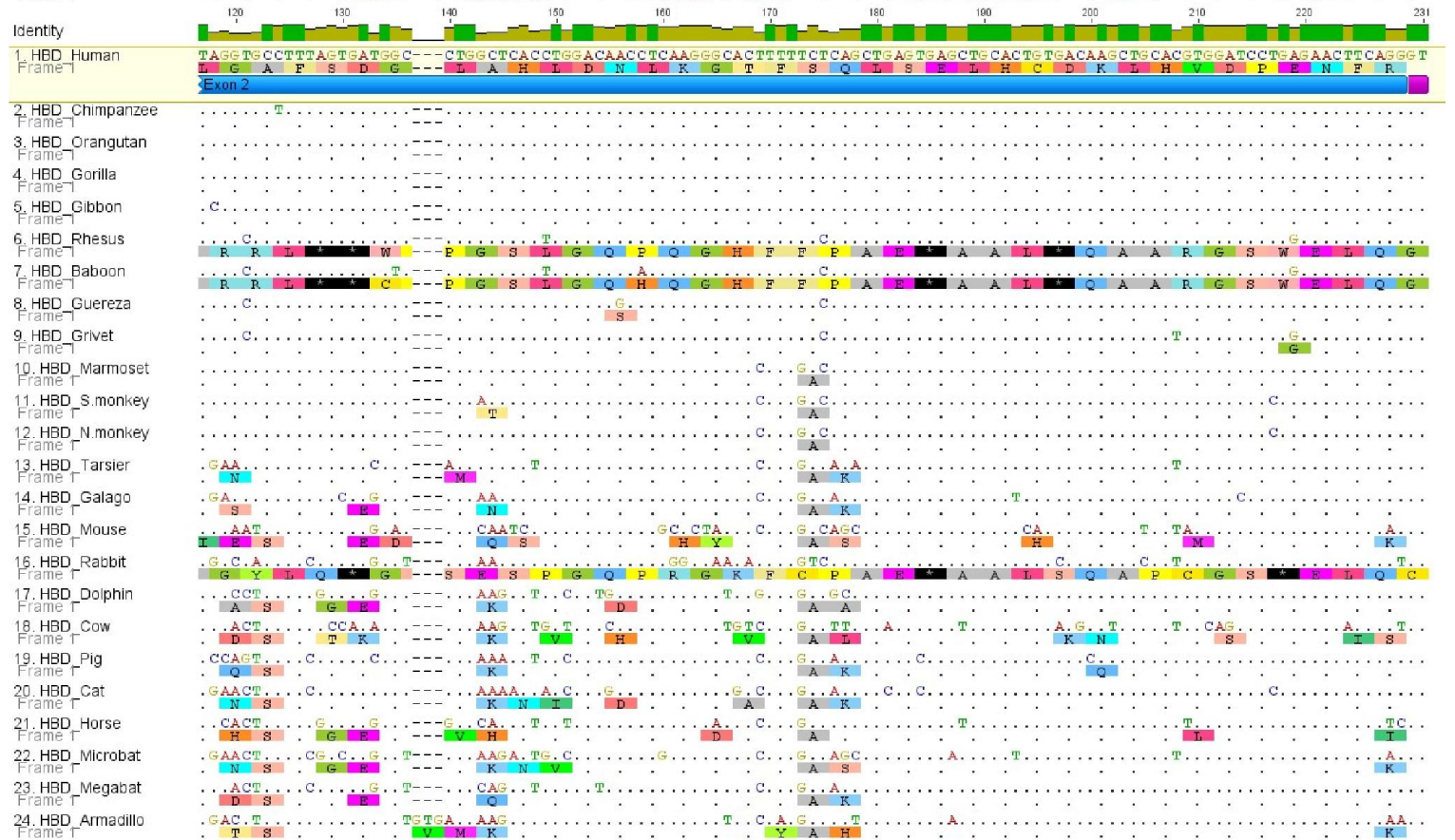

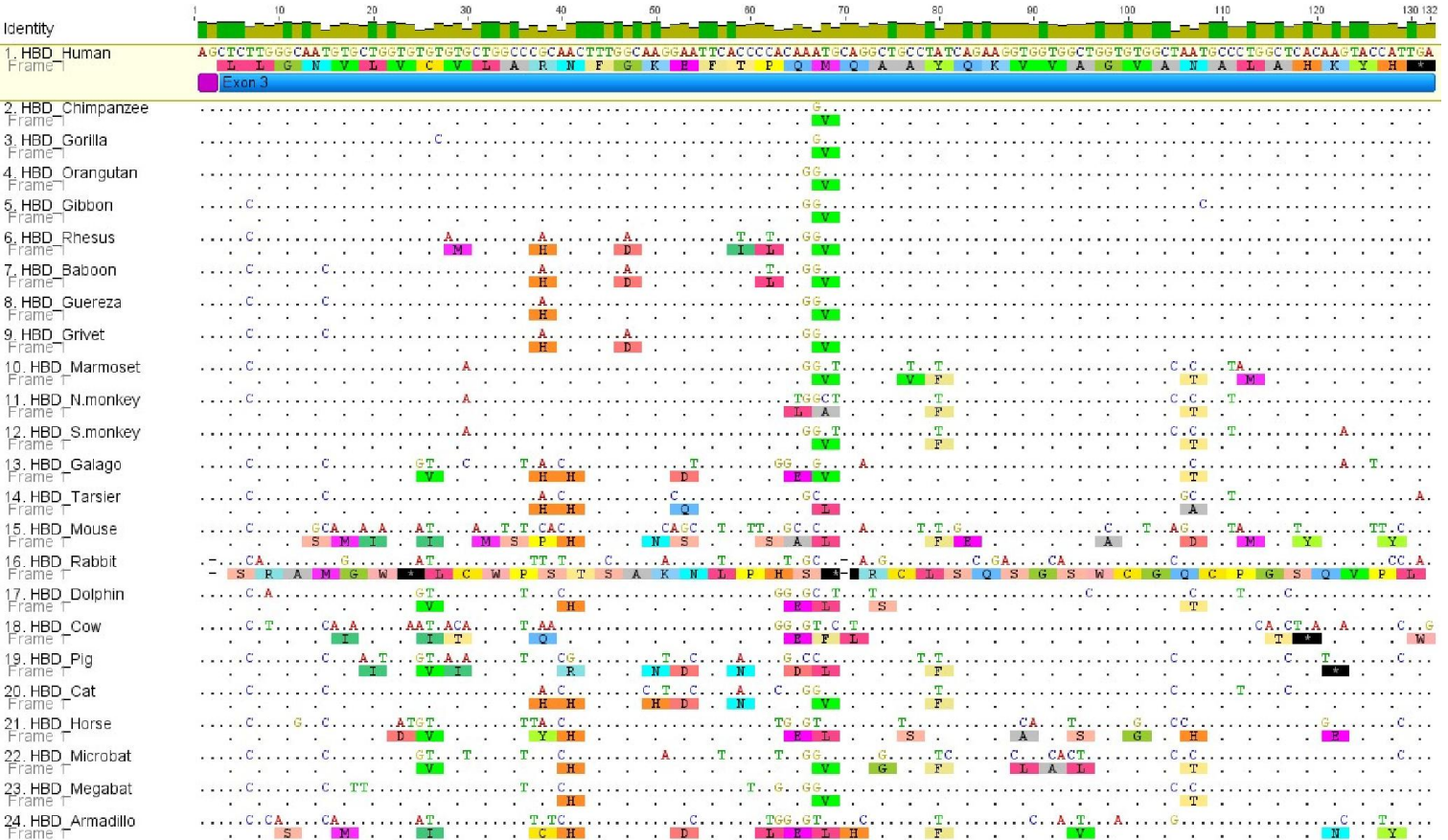

Supplement: S2 Fig — Blue and purple filled boxes mark the exons and donnor/acceptor splice sites, respectively. Dots represent nucleotide identities to the human sequence that was set as reference. Coloured nucleotides indicate changes to the human sequence and aminoacid alterations are marked by filled coloured boxes. The lemur species were excluded from the analysis given that their hybrid ψβ/δ pseudogene [37] generates multiple misalignments. (PDF) [file pone.0123365.s002.pdf]

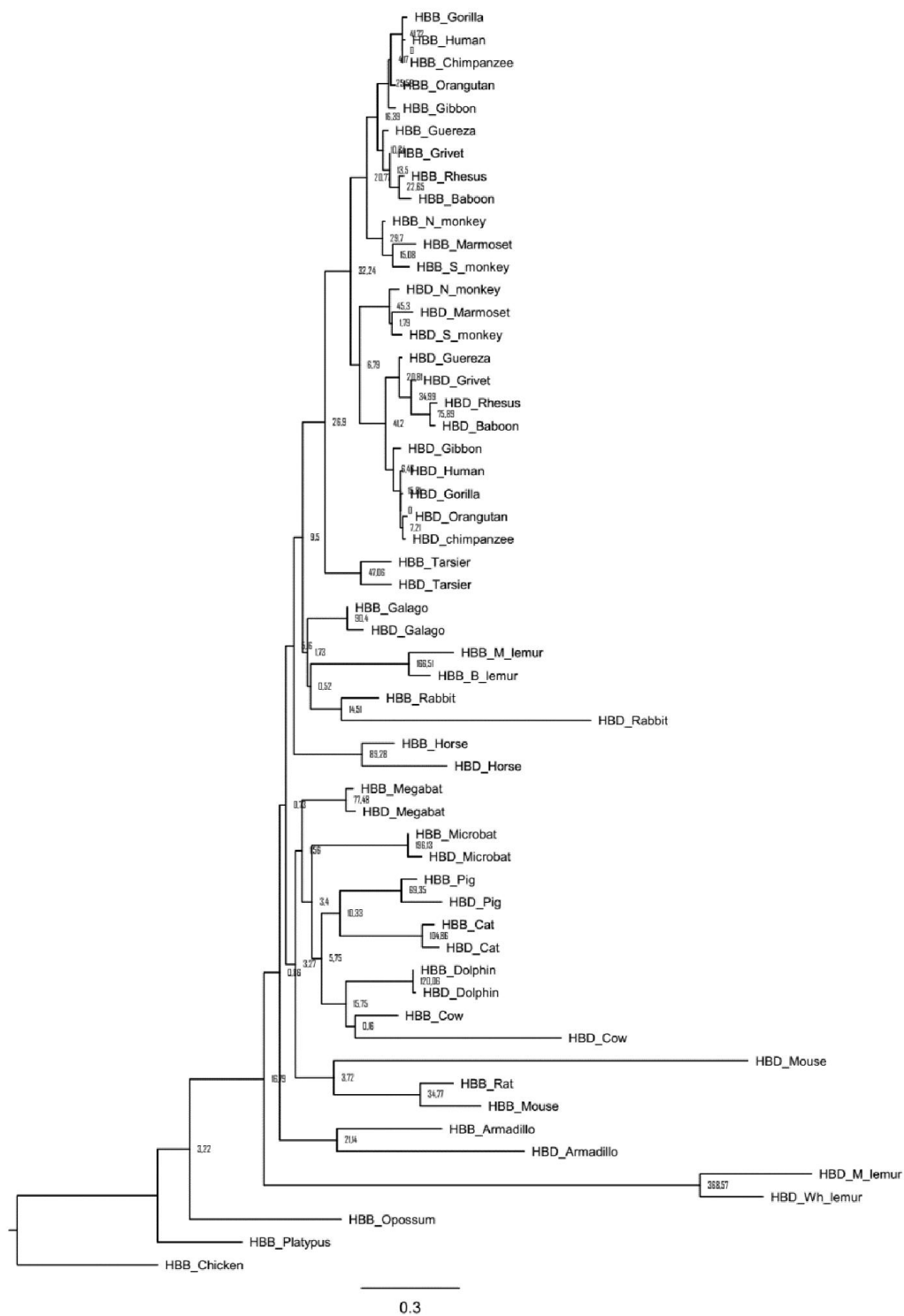

Supplement: S4 Fig — The phylogenic tree, based on the coding sequence, was constructed using the Goldman–Yang codon model. Branch support values, obtained using the approximate Likelihood Ration Test (aLRT), are given on the internodes. (PDF) [file pone.0123365.s004.pdf]

A

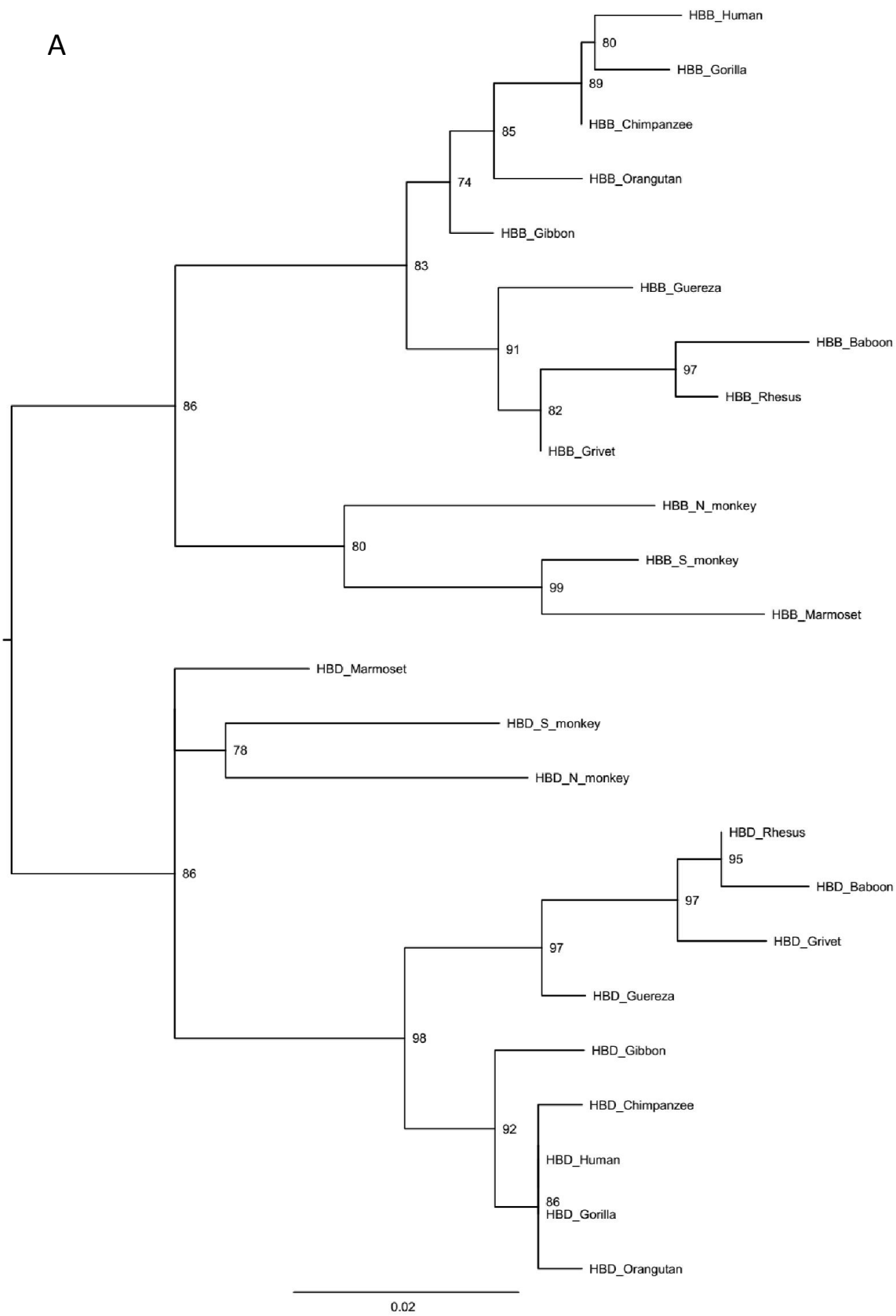

B

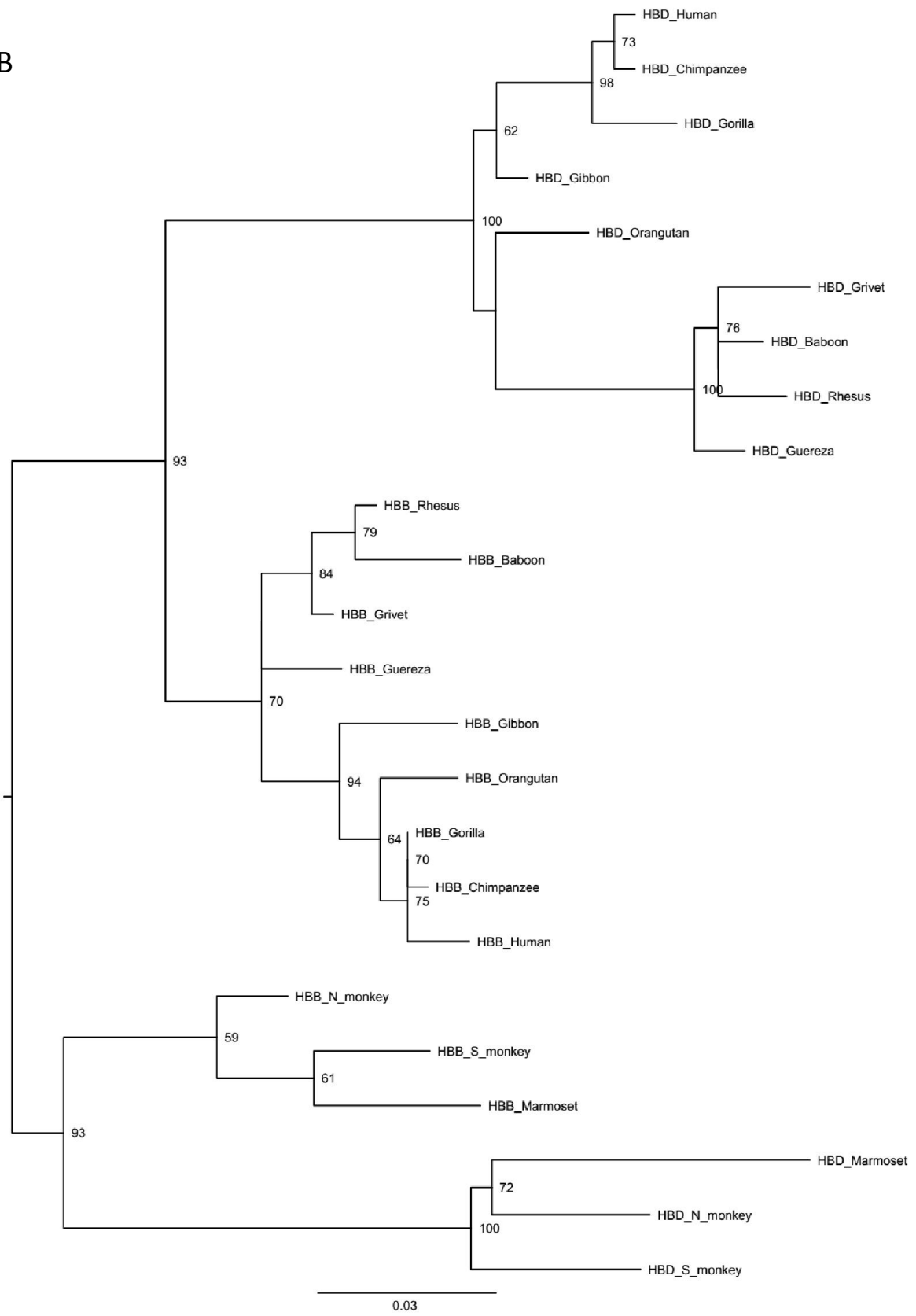

Supplement: S5 Fig — The phylogeny reconstructions were based on A) the portion of the alignment that contain evidence of the anthropoid gene conversion (nucleotide 367–632) and B) the portion of the alignment between the inferred breakpoint in event 3 (nucleotide 97–385). Bootstrap branch support (1000 replicates) are given on the internodes. (PDF) [file pone.0123365.s005.pdf]
