# Supplementary material for: Distinctive Patterns of Evolution of the δ-Globin Gene (HBD) in Primates
Source: PLoS One. 2015 Apr 8;10(4):e0123365. doi: 10.1371/journal.pone.0123365 (PMC4390247; doi:10.1371/journal.pone.0123365)
Supplement: S3 Fig — A) HBB and HBB-like HBD promoters; B) Anthropoid HBD promoters and C) HBD promoters lacking the TF binding motifs which are conserved in HBB-like and HBD-like promoters. Conserved binding motifs are indicated in grey boxes. Again, the lemur species were excluded from the analysis. (PDF) [file pone.0123365.s003.pdf]

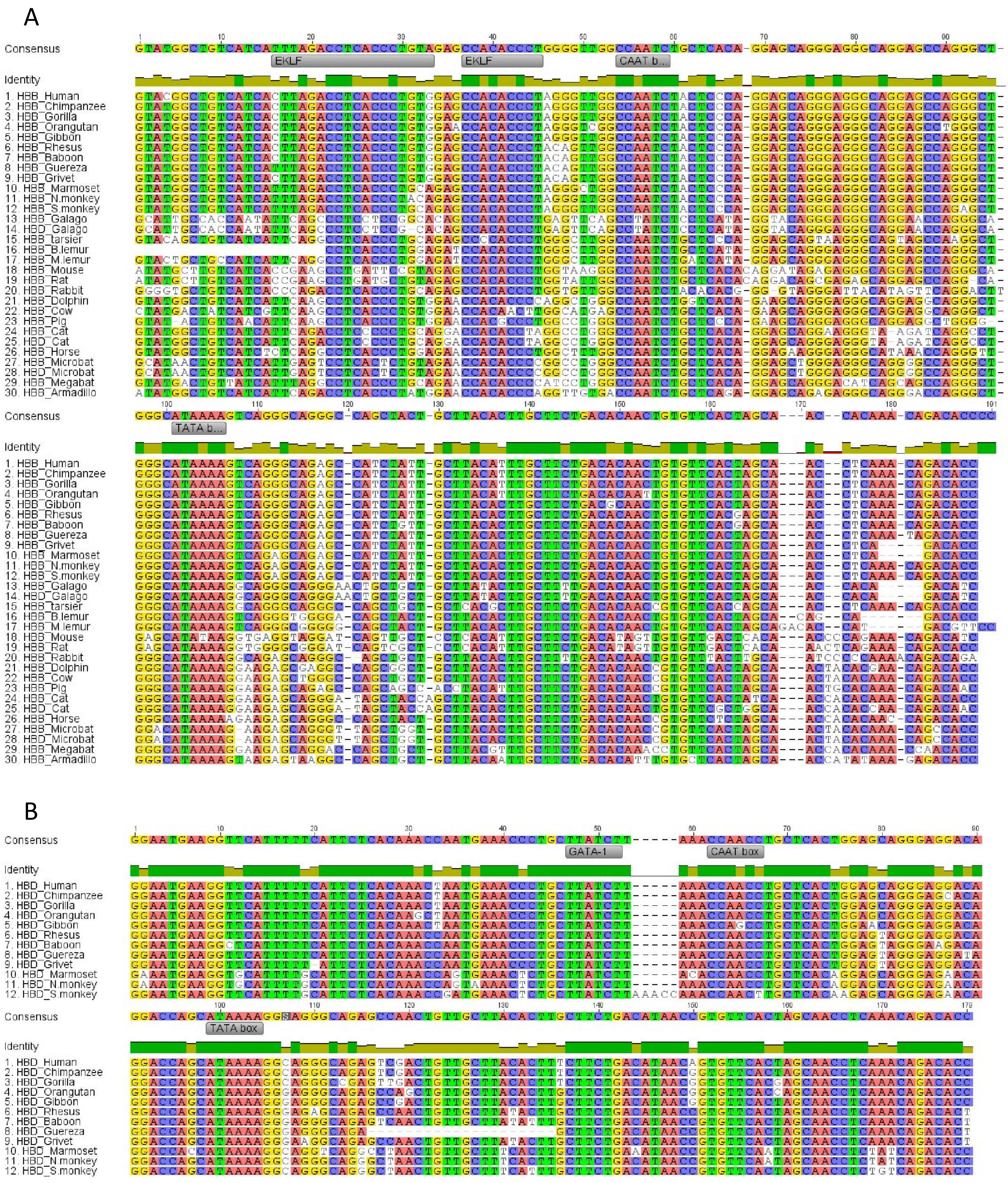
A
Consensus
EKLF
EKLF
CAAT b...
Identity
1. HBB_Human
2. HBB_Chimpanzee
3. HBB_Gorilla
4. HBB_Orangutan
5. HBB_Gibbon
6. HBB_Rhesus
7. HBB_Baboon
8. HBB_Guereza
9. HBB_Grivet
10. HBB_Marmoset
11. HBB_N.monkey
12. HBB_S.monkey
13. HBB_Galago
14. HBD_Galago
15. HBB_tarsier
16. HBB_B.lemur
17. HBB_M.lemur
18. HBB_Mouse
19. HBB_Rat
20. HBB_Rabbit
21. HBB_Dolphin
22. HBB_Cow
23. HBB_Pig
24. HBB_Cat
25. HBD_Cat
26. HBB_Horse
27. HBB_Microbat
28. HBD_Microbat
29. HBB_Megabat
30. HBB_Armadillo
TATA b...
B
GATA-1
CAAT box
1. HBD_Human
2. HBD_Chimpanzee
3. HBD_Gorilla
4. HBD_Orangutan
5. HBD_Gibbon
6. HBD_Rhesus
7. HBD_Baboon
8. HBD_Guereza
9. HBD_Grivet
10. HBD_Marmoset
11. HBD_N.monkey
12. HBD_S.monkey
TATA box

C

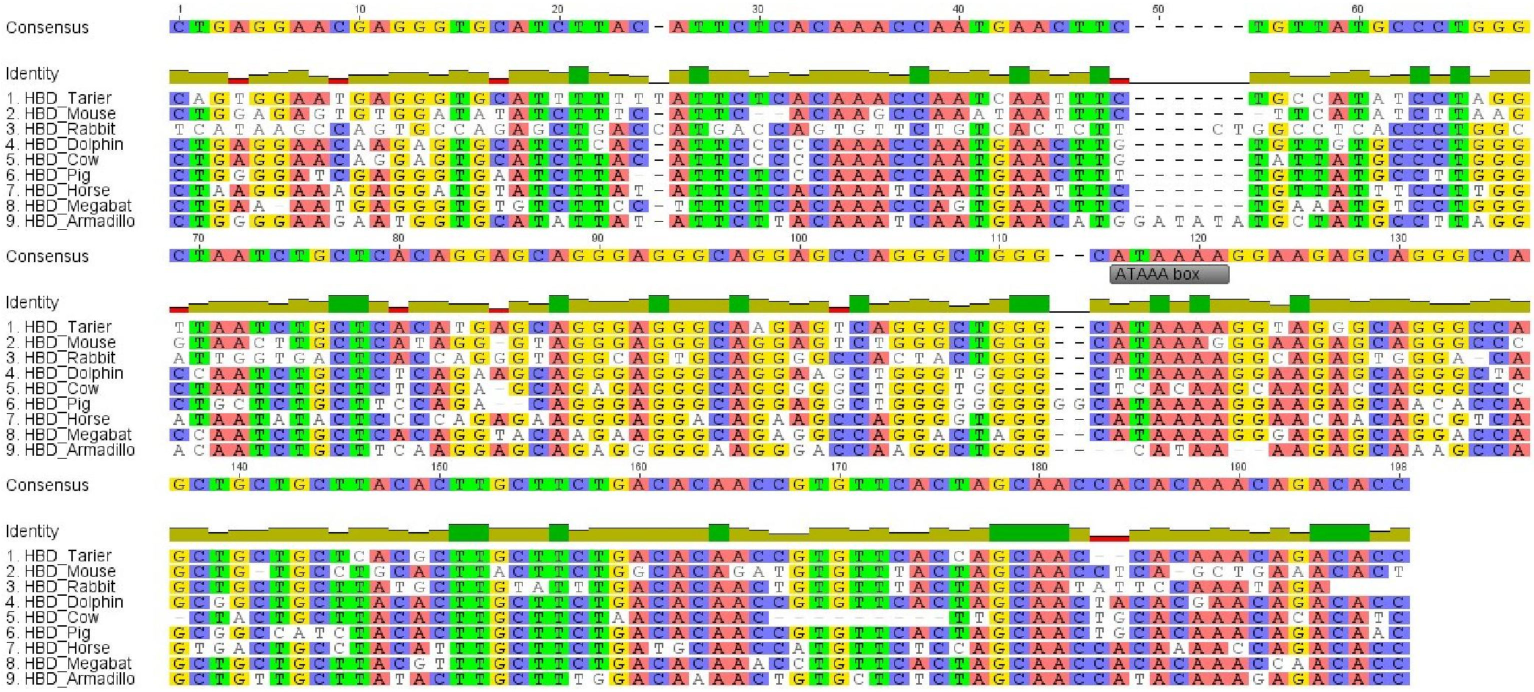
Consensus
Identity
1. HBD_Tarier
2. HBD_Mouse
3. HBD_Rabbit
4. HBD_Dolphin
5. HBD_Cow
6. HBD_Pig
7. HBD_Horse
8. HBD_Megabat
9. HBD_Armadillo
ATAAA box
