# Supplementary material for: Distinctive Patterns of Evolution of the δ-Globin Gene (HBD) in Primates
Source: PLoS One. 2015 Apr 8;10(4):e0123365. doi: 10.1371/journal.pone.0123365 (PMC4390247; doi:10.1371/journal.pone.0123365)
Supplement: S1 Table — (PDF) [file pone.0123365.s006.pdf]

| Order | SCIENTIFIC NAME | COMMON NAME | Assembly/ Accession Number | HBB | HBD |
|---|---|---|---|---|---|
| Primates | *Homo sapiens** | Human | GRCh37/hg19 | chr11:5246600-5248430 | chr11:5253981-5255837 |
| | *Pan troglodytes** | Chimpanzee | CSAC 2.1.4/panTro4 | chr11:4976349-4978179 | chr11:4983729-4985583 |
| | *Gorilla gorilla** | Gorilla | gorGor3.1/gorGor3 | chr11:5181180-5183419 | chr11:5189200-5190818 |
| | *Pongo pygmaeus** | Orangutan | WUGSC 2.0.2/ponAbe2 | chr11:65237578-65239448 | chr11:65230079-65231933 |
| | *Nomascus leucogenys* | Gibbon | GGSC Nleu3.0/nomLeu3 | chr15:67092208-67094052 | chr15:67099627-67101481 |
| | *Papio anubis* | Baboon | Baylor Panu_2.0/papAnu2 | chr14:60046909-60048748 | chr14:60039529-60041379 |
| | *Macaca mulatta** | Rhesus | BGI CR_1.0/rheMac3 | chr14:68486458-68488296 | chr14:68479060-68480908 |
| | *Colobus guereza* | Guereza | AC175618.2 | Range:43444-45290 | Range:50789-52640 |
| | *Chlorocebus aethiops* | Grivet | AC192680.2 | Range:56560-58450 | Range:64005-65870 |
| | *Callithrix jacchus* | Marmoset | WUGSC 3.2/calJac3 | chr11:68655845-68657643 | chr11:68662011-68663826 |
| | *Aotus nancymaae* | Night monkey | AC174399.2 | Range:86520-88480 | Range:93020-94850 |
| | *Saimiri boliviensis* | Squirrel monkey | Broad/saiBol1 | JH378113:894229-896044 | JH378113:887920-889755 |
| | *Microcebus murinus* | Mouse lemur | Broad/micMur1 | scaffold_23051:7558-9396 | scaffold_23051:11861-13574 |
| | *Eulemur macaco* | Brown lemur | - | M15734.1 | - |
| | *Eulemur albifrons* | White-headed lemur | - | - | V00644.1 |
| | *Tarsius syrichta* | Tarsier | Broad/tarSyr1 | J04429.1 | scaffold_30223:10672-12718 |
| | *Otolemur garnettii* | Galago | U60902.1 | Range:50897-53130 | Range:46123-48125 |
| Rodentia | *Rattus norvegicus* | Rat | Baylor 3.4/rn4 | chr1:161618682-161620327 | - |
| | *Mus musculus* | Mouse | GRCm38/mm10 | chr7:103826432-103828057 | chr7:103838921-103840610 |
| Lagomorpha | *Oryctolagus cuniculus* | Rabbit | Broad/oryCun2 | chr1:146236925-146238433 | chr1:146245465-146247150 |
| Carnivora | *Felis catus* | Cat | AC129072.3 | Range:29610-31430 | Range:33950-35770 |
| Chiroptera | *Myotis lucifugus* | Microbat | Myoluc2.0/myoLuc2 | GL429905:2514138-2515941 | GL429905:2519498-2521193 |
| | *Pteropus vampyrus* | Megabat | Broad/pteVam1/<br><br>AC216164.2 | scaffold_3459:3976-2676<br><br>Range:179400-179563 | scaffold_3459:9145-11021 |
| Cetartiodactyla | *Bos Taurus* | Cow | Baylor Btau_4.6.1/bosTau7 | chr15:47792823-47794689 | chr15:47782257-47784127 |
| | *Sus scrofa* | Pig | SGSC Sscrofa10.2/susScr3 | chr9:5632884-5634490 | chr9:5640805-5642408 |

| | | | | | |
|---|---|---|---|---|---|
| | *Tursiops truncatus* | Dolphin | Baylor Ttru_1.4/turTru2<br><br>gnl\|ti\|2241352997<br><br>XM_004330598.1 | JH496320:15248-16841<br><br>Range:366-500 | JH496320:8718-10536 |
| Perissodactyla | *Equus caballus* | Horse | Broad/equCab2 | chr7:73936358-73937959 | chr7:73943584-73945347 |
| Cingulata | *Dasypus novemcinctus* | Armadillo | Baylor/dasNov3 | JH576106:328717-330317 | JH576106:324817-326487 |
| Didelphimorphia | *Monodelphis domestica* | Opossum | Broad/monDom5 | chr4:352263811-352265844 | - |
| Monotremata | *Ornithorhynchus anatinus* | Platypus | WUGSC 5.0.1/ornAna1 | Contig7843:24151-25617 | - |
| Galliformes | *Gallus gallus* | Chicken | ICGSC Gallus_gallus-4.0/galGal4 | chr1:193728454-193730403 | - |

*sequences confirmed by sequencing
