# Supplementary material for: Distinctive Patterns of Evolution of the δ-Globin Gene (HBD) in Primates
Source: PLoS One. 2015 Apr 8;10(4):e0123365. doi: 10.1371/journal.pone.0123365 (PMC4390247; doi:10.1371/journal.pone.0123365)
Supplement: S2 Table — (PDF) [file pone.0123365.s007.pdf]

	Time (Myr)	Exons			Introns		
		HBB	***HBD***	***HBBP1***	***HBB***	***HBD***	***HBBP1***
Hum-Ptr	6,3	0,18	0,54	0,90	1,39	0,94	1,15
Hum-Ggo	8,8	0,26	0,26	0,52	0,88	0,90	1,18
Hum-Ppy	15,7	0,58	0,29	1,18	1,13	1,08	1,71
Hum-Nle	20,4	0,56	0,39	1,15	0,98	1,13	1,40
Hum-Mcc	29	0,80	0,88	1,18	1,25	0,89	1,53
Hum-Panu		0,84	0,92	1,18	1,29	0,86	1,61
Hum-Cgue		0,55	0,55	1,48	1,24	1,08	1,67
Hum-Caa		0,55	0,80	1,14	1,35	1,04	1,71
Hum-Sbol	42,6	0,80	0,83	1,39	1,75	1,30	1,54
Hum-Cjac		0,74	0,98	1,54	1,77	1,53	1,38
Hum-Anan		0,60	0,80	1,44	1,56	1,38	1,36
Hum-Tsyr	65,2	0,92	0,92	1,66	2,54	2,94	2,77
Hum-Ogar	74	0,74	0,82	4,66	2,78	5,41	3,92
